# Quantitative and Longitudinal Assessment of Systemic Innate Immunity in Health and Disease Using a 2D Gene Model

**DOI:** 10.3390/biomedicines12050969

**Published:** 2024-04-27

**Authors:** Hongxing Lei

**Affiliations:** 1 CAS Key Laboratory of Genome Sciences and Information, Beijing Institute of Genomics, Chinese Academy of Sciences, and China National Center for Bioinformation, Beijing 100101, China; leihx@big.ac.cn; Tel.: +86-010-8409-7276; 2 Cunji Medical School, University of Chinese Academy of Sciences, Beijing 101408, China

**Keywords:** innate immunity, viral infection, bacterial infection, host response, accessible health monitoring

## Abstract

Dysregulation of innate immunity is deeply involved in infectious and autoimmune diseases. For a better understanding of pathogenesis and improved management of these diseases, it is of vital importance to implement convenient monitoring of systemic innate immunity. Built upon our previous works on the host transcriptional response to infection in peripheral blood, we proposed a 2D gene model for the simultaneous assessment of two major components of systemic innate immunity, including VirSig as the signature of the host response to viral infection and BacSig as the signature of the host response to bacterial infection. The revelation of dysregulation in innate immunity by this 2D gene model was demonstrated with a wide variety of transcriptome datasets. In acute infection, distinctive patterns of VirSig and BacSig activation were observed in viral and bacterial infection. In comparison, both signatures were restricted to a defined range in the vast majority of healthy adults, regardless of age. In addition, BacSig showed significant elevation during pregnancy and an upward trend during development. In tuberculosis (TB), elevation of BacSig and VirSig was observed in a significant portion of active TB patients, and abnormal BacSig was also associated with a longer treatment course. In cystic fibrosis (CF), abnormal BacSig was observed in a subset of patients, and no overall change in BacSig abnormality was observed after the drug treatment. In systemic sclerosis-associated interstitial lung disease (SSc-ILD), significant elevation of VirSig and BacSig was observed in some patients, and treatment with a drug led to the further deviation of BacSig from the control level. In systemic lupus erythematosus (SLE), positivity for the anti-Ro autoantibody was associated with significant elevation of VirSig in SLE patients, and the additive effect of VirSig/BacSig activation was also observed in SLE patients during pregnancy. Overall, these data demonstrated that the 2D gene model can be used to assess systemic innate immunity in health and disease, with the potential clinical applications including patient stratification, prescription of antibiotics, understanding of pathogenesis, and longitudinal monitoring of treatment response.

## 1. Introduction

Innate immunity lies on the front line of the host defense against pathogen invasion [1]. Apart from acute infection, innate immunity is also deeply involved in chronic infection and autoimmune diseases [2]. Thus, convenient assessment of innate immunity has broad applications in clinical settings as well as health monitoring. Currently, a diverse set of immune and inflammation markers have been routinely utilized in medical institutions around the globe [3]. However, the high heterogeneity of many infectious and autoimmune diseases presents great challenges to the understanding of the pathogenesis and treatment response [4,5]. It would be desirable to have novel markers that are more informative of the innate immunity in the host defense mechanism.

Gene dysregulation in peripheral blood has been investigated in transcriptome studies on the host defense related to infection [6]. Our previous computational analysis of multiple independent transcriptome datasets revealed consensus gene dysregulation in response to viral or bacterial infection [7]. These consensus genes were mainly involved in two of the well-known host defense pathways, namely the interferon signaling pathway and neutrophil degranulation [8,9]. Thereafter, we have also used an RT-PCR platform to confirm many of these consensus genes using hundreds of clinical samples [10,11]. With the fast transition from microarray (analog) to RNA-Seq (digital) platforms in recent years, a higher resolution of gene expression measurement has been achieved. Thus, we were wondering whether the improved resolution could lead to a quantitative gene model for the assessment of innate immunity in relevant situations.

In the current work, a 2D gene model reflecting the host response to viral and bacterial infection (five genes each) was proposed as a marker of innate immunity, and selected diseases with a close connection to innate immunity were investigated. For acute infection, the distinctions in innate immunity were examined between viral and bacterial infection, active infection and inactive colonization, acute phase and convalescence phase, and patients with post-acute sequelae (PAS) and non-infected controls. As a comparison, the innate immunity in healthy subjects was also examined in adulthood as well as during development and pregnancy. For chronic infection and autoimmune diseases, tuberculosis, cystic fibrosis and SSc-ILD were examined for the prevalence of dysregulation in innate immunity and the heterogeneous response to the respective drug treatment. In addition, SLE was examined for the correlation of innate immunity with a certain autoantibody and the additive effect of immune dysregulation in pregnancy. Throughout this work, it was demonstrated that the 2D gene model was suitable for the quantitative and longitudinal assessment of innate immunity in relevant conditions. The small size of the gene panel makes it convenient to simultaneously assess two major pathways of innate immunity. The potential clinical applications will be discussed.

## 2. Materials and Methods

All of the transcriptome datasets analyzed in this work were downloaded from the Gene Expression Omnibus (GEO, https://www.ncbi.nlm.nih.gov/geo/, accessed on 20 March 2024). For consistency, the tissue source was restricted to whole blood for all of these datasets. The selection criteria also required the inclusion of healthy controls in every dataset. There were a total of 17 transcriptome datasets and 2168 samples included in this work. The transcriptome experiments were conducted on RNA-Seq (RNA sequencing) platforms. The raw gene counts were normalized and log2 transformed for the downstream analyses. The expression values of the signature genes were extracted from these datasets. The average expression values of the VirSig and BacSig genes were calculated and normalized based on the reference VirSig and BacSig values. The statistical calculations was performed in R (https://www.r-project.org/, accessed on 20 March 2024). The two-group comparisons were performed using *t*-test (paired *t*-test for the same people comparison and unpaired *t*-test for the case/control comparison). The multi-group comparisons were performed using one-way ANOVA followed by Tukey’s test (Tukey’s Honest Significant Differences). The figures were drawn using the ggplot2 package in R.

### 2.1. Analysis of VirSig and BacSig in All Phases of Acute Infection

Six transcriptome datasets were used for the analysis of VirSig and BacSig in different phases of acute infection. Two of those datasets were related to the acute phase of infection, including both viral and bacterial infection in each dataset (GSE161731 and GSE176262) [12,13]. One dataset was related to the comparison of active infection and inactive colonization of group A streptococcal (GSE158163) [14]. Another dataset was related to the comparison of the acute phase and convalescence phase of Leptospirosis (GSE72946) [15]. The remaining two datasets were related to the long-term effect of acute infection, including one on Ebola survivors (GSE143549) [16] and the other on chronic fatigue syndrome (GSE98139) [17].

### 2.2. Analysis of VirSig/BacSig in a Healthy Population

Four transcriptome datasets were used for the analysis of VirSig and BacSig in a healthy population. One of these datasets was related to the age effect in healthy adults (GSE186507) [18]. Another dataset was related to various stages of normal development (GSE231409). The third dataset was related to the gender effect in healthy adults (GSE134080) [19]. The final dataset was related to various stages of normal pregnancy (GSE108497) [20].

### 2.3. Analysis of VirSig/BacSig in Chronic Infection

Four transcriptome datasets were used for the analysis of VirSig and BacSig in chronic infection. One of these datasets was related to the comparison of active TB with latent TB as well as the comparison among various subtypes of active TB (GSE107995) [21]. Two datasets were related to the treatment response in TB (GSE157657 and GSE89403) [22,23]. The other dataset was related to the treatment response in cystic fibrosis (GSE124548) [24].

### 2.4. Analysis of VirSig/BacSig in Autoimmune Diseases

Three transcriptome datasets were used for the analysis of VirSig and BacSig in autoimmune diseases. One of these datasets was related to the treatment response in SSc-ILD (GSE181228) [25]. The other two datasets were related to SLE, including one on the autoantibody (GSE72509) [26] and the other on pregnancy (GSE235508) [27].

## 3. Results

For convenient assessment of innate immunity, two small gene sets for the signatures of the host transcriptional response to infection were selected based on our previous analysis of microarray data. VirSig was defined as the signature of the host response to viral infection using the average expression of five representative genes, including *IFI27*, *RSAD2*, *IFI44L*, *ISG15* and *IFITM3*. BacSig was defined as the signature of the host response to bacterial infection using the average expression of five representative genes, including *S100A12*, *CD177*, *HP*, *ANXA3*, and *ARG1*. For every dataset examined in this work (Table 1), the reference values of VirSig and BacSig were first calculated. The reference values were defined as the median VirSig and BacSig values of the control group. For easier comparison across different datasets, the VirSig and BacSig values of every sample were subtracted by the references values, which indicated the deviation from the reference point. The statistical analyses were based on the normalized VirSig and BacSig values. The *p*-values from the statistical analyses can be found in the Supplementary Materials (Supplementary Table S1).

### 3.1. Features of VirSig and BacSig Activation in Acute Infection

**Figure 1:**
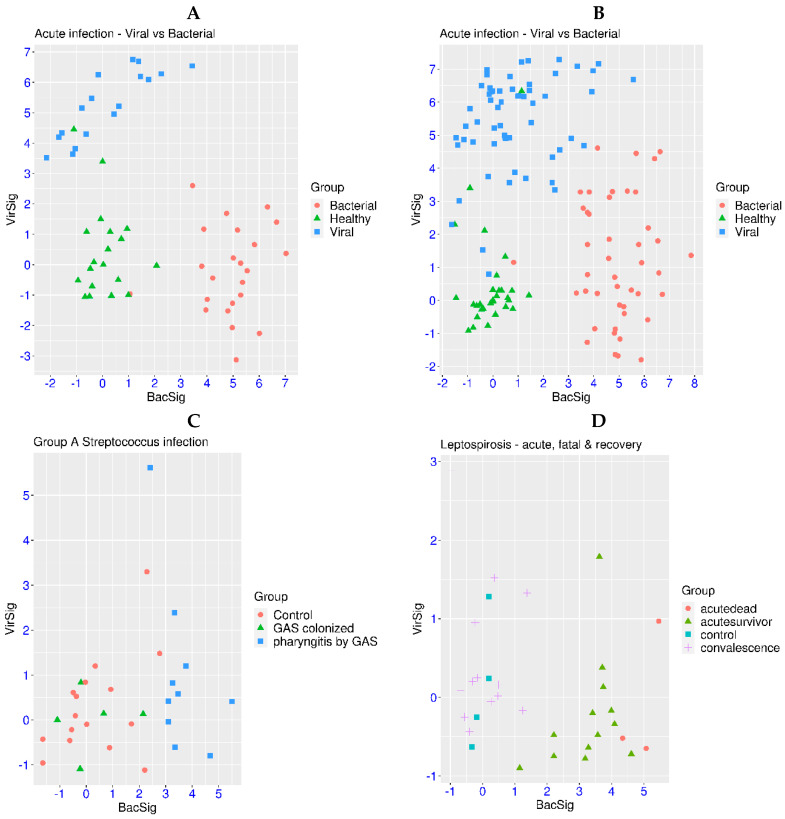

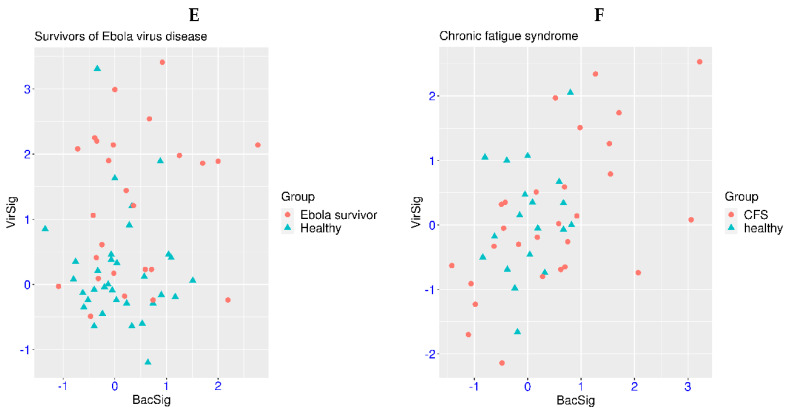
Patterns of VirSig and BacSig activation in acute infection. (**A**) Top left, distinctive activation of VirSig and BacSig in the acute phase of viral or bacterial infection (GSE161731). (**B**) Top right, distinctive activation of VirSig and BacSig in the acute phase of viral or bacterial infection in another dataset (GSE176262). (**C**) Middle left, distinctive activation of BacSig in active infection (pharyngitis) or inactive colonization of group (**A**) *Streptococcus* (GSE158163). (**D**) Middle right, distinctive activation of BacSig at the acute or convalescent stage of Leptospirosis (GSE72946). The outcome of the patients at the acute phase was also indicated (dead or survivor). (**E**) Bottom left, persistent activation of VirSig and BacSig in some survivors of Ebola virus disease (GSE143549). (**F**) Bottom right, persistent activation of BacSig in some patients with chronic fatigue syndrome (GSE98139).

First, the features of VirSig and BacSig activation at different stages of acute infection were examined using six relevant transcriptome datasets. In dataset GSE161731, patients with viral or bacterial infection were compared with healthy controls. It was evident that samples from the healthy control group generally resided in the lower left corner of the BacSig/VirSig 2D graph (BacSig < 2 and VirSig < 3, Figure 1A). A few outliers were observed in the control group. One healthy subject had a BacSig slightly over 2, while two healthy subjects had a VirSig > 3. In comparison, all of the patients with viral infection had a VirSig > 3, and all but one patient with bacterial infection had a BacSig > 3. The *p*-values were <1.0 × 10^−7^ for the comparison of the VirSig in the viral infection group against the VirSig in either the bacterial infection group or the healthy control group. The *p*-values were also <1.0 × 10^−7^ for the comparison of the BacSig in the bacterial infection group against the BacSig in either the viral infection group or the healthy control group. On the other hand, the *p*-value was >0.05 for the comparison of the VirSig in the bacterial infection group and the healthy control group. The *p*-value was also >0.05 for the comparison of the BacSig in the viral infection group and the healthy control group. This clearly demonstrated that VirSig and BacSig were bona fide signatures of the host transcriptional response to viral and bacterial infection, respectively. 

Similar features were observed in another dataset on viral and bacterial infection (Figure 1B, GSE176262). None of the healthy subjects had a BacSig > 2, while only two healthy subjects had a VirSig > 3. In comparison, all but three patients with viral infection had a VirSig > 3, and all but one patient with bacterial infection had a BacSig > 3. The *p*-values were <1.0 × 10^−7^ for the comparison of the VirSig in the viral infection group against the VirSig in either the bacterial infection group or the healthy control group. The *p*-values were also <1.0 × 10^−7^ for the comparison of the BacSig in the bacterial infection group against the BacSig in either the viral infection group or the healthy control group. On the other hand, the *p*-value was >0.05 for the comparison of the VirSig in the bacterial infection group and healthy control group. In addition, the *p*-value was 0.004 for the comparison of the BacSig in the viral infection group and the healthy control group. Overall, highly consistent patterns were observed in these two datasets on acute infection. The healthy controls generally had a BacSig < 2 and a VirSig < 3. Patients with bacterial infection mostly had a BacSig > 3, while patients with viral infection mostly had a VirSig > 3. Thus, distinctive activation of innate immunity in viral or bacterial infection could be revealed with the simultaneous assessment of the VirSig and BacSig. 

For patients with fever, positive culture is one of the major considerations for the prescription of antibiotics. However, false positives can arise when the detected bacteria are inactive and the symptom is due to other factors. The distinction between active infection and inactive colonization of group A *Streptococcus* (GAS) was studied in a related dataset (GSE158163). In this dataset, the minimum BacSig value was 2.41 in the active infection group, while the maximum BacSig value was 2.15 in the inactive colonization group (Figure 1C). In addition, nine of the ten patients in the active infection group had a BacSig > 3. The *p*-values were <6.4 × 10^−5^ for the comparison of the BacSig in the active infection group against the BacSig in either the inactive colonization group or the healthy control group. On the other hand, the *p*-values were >0.05 for the BacSig comparison between the inactive colonization group and the healthy control group. The *p*-values were also >0.05 for the VirSig comparison among the three groups. This indicated that the BacSig and VirSig in the inactive colonization group were indistinguishable from those in the healthy control group. Thus, BacSig/VirSig could be used to assist the discrimination of active infection from inactive colonization and reduce false positives.

For acute infection, it is also important to know whether innate immunity is restored to the pre-infection state when the patients are recovered from the acute phase. In a related dataset on Leptospirosis (GSE72946), acute infection was compared against the recovery phase. First, the VirSig values were <3 in all of the samples in the different groups (Figure 1D), indicating non-activation of VirSig in this infection (*p* > 0.05). On the other hand, the *p*-value was <1.0 × 10^−7^ for the BacSig comparison between the acute infection group and the convalescence group. The maximum BacSig value was 1.38 in the convalescence group, while all but one patient had a BacSig > 2 in the acute infection group. This demonstrated the restoration of the BacSig to the pre-infection level in the recovered patients. Moreover, the 3 patients with bad outcomes all had a BacSig > 4.30, while 12 of the 13 survivors had a BacSig < 4.30 in the acute infection phase. Thus, BacSig/VirSig could assist patient prognosis in the acute infection phase and the assessment of the restoration of innate immunity in the convalescence phase [28,29].

For some patients, lingering health conditions can persist long after the acute infection phase, which is termed post-acute sequelae [30]. Abnormal immunity is suspected for subjects with PAS. In a related dataset on Ebola survivors two years after discharge from the treatment center (GSE143549), survivors of Ebola virus disease were compared against uninfected controls. All of the healthy subjects had a BacSig < 2, and only one healthy subject had a VirSig > 3 (Figure 1E). In comparison, three Ebola survivors had a BacSig > 2, and one Ebola survivor had a VirSig > 3. This indicated persistent abnormal BacSig values in a small portion of Ebola survivors. In addition, 8 of the 26 Ebola survivors (30.8%) had a VirSig > 2, while only 1 of the 33 healthy subjects (3.0%) had a VirSig > 2. The *p*-values were 4.8 × 10^−4^ and 0.22 for the VirSig and BacSig comparison between the two groups, respectively. The mean difference in the VirSig for the two groups was 1.0, indicating a significant upward shift in the VirSig in Ebola survivors. Thus, VirSig/BacSig could reveal the persistent abnormality of innate immunity in PAS, both at the group level and at the individual level.

As another type of PAS, many of the patients with chronic fatigue syndrome (CFS) are suspected to have previous infection as the source of the complex health problems [31]. In a related dataset (GSE98139), CFS patients were compared against healthy controls. In this dataset, all of the samples had a VirSig < 3 and the *p*-value was 0.92 for the VirSig comparison between the two groups (Figure 1E), indicating the non-existence of persistent VirSig abnormality in the CFS patients. On the other hand, all of the healthy subjects had a BacSig < 1, while 7 of the 28 CFS patients (25.0%) had a BacSig > 1 and 3 patients had a BacSig > 2. Although statistical significance was not reached for the BacSig comparison between the two groups (*p* = 0.079), the disproportional distribution of samples with a BacSig > 1 might still indicate the persistent abnormality of innate immunity in some CFS patients. It should be noted that the pattern of persistent abnormality was different in the two types of PAS examined here. The abnormality was mainly reflected in the VirSig for Ebola survivors, while it was mainly reflected in the BacSig for CFS patients. Thus, simultaneous assessment of the VirSig and BacSig is highly recommended for patients with PAS.

### 3.2. VirSig/BacSig Features in Healthy Population: Age, Sex and Pregnancy

**Figure 2:**
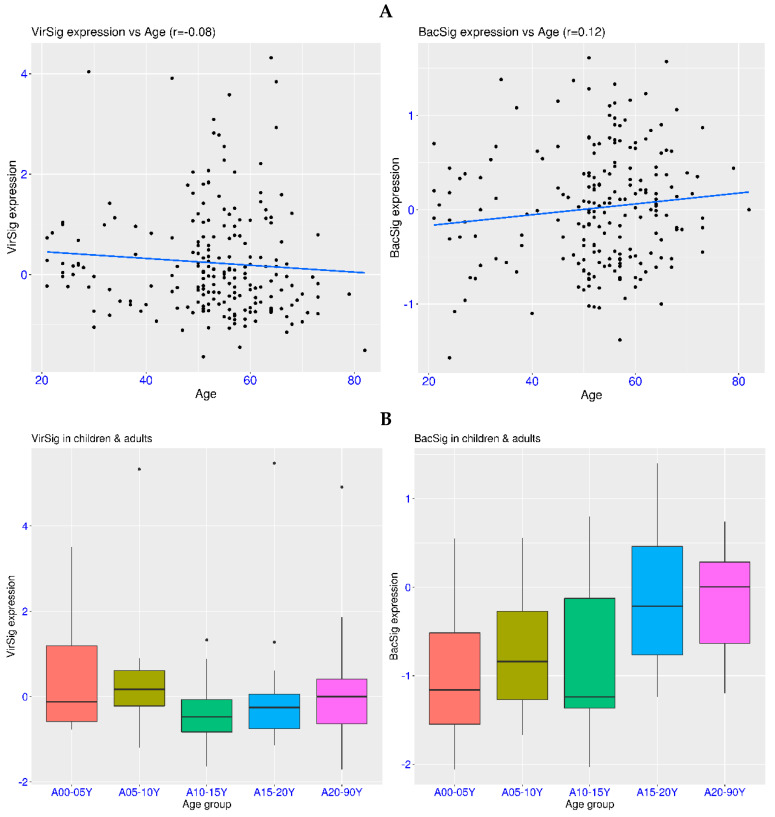

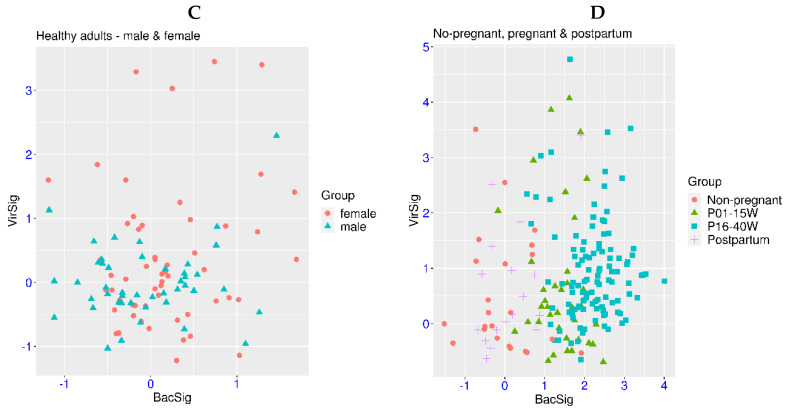
VirSig/BacSig features in healthy population. (**A**) Top panel, correlation of baseline VirSig or BacSig value with age among healthy adults (GSE186507). The blue line indicated linear regression. (**B**) Middle panel, baseline VirSig and BacSig values in different age groups (GSE231409). The age groups included 0–5 years old (A00–05Y), 5–10 years old (A05–10Y), 10–15 years old (A10–15Y), 15–20 years old (A15–20Y), and 20–90 years old (A20–90Y). (**C**) Bottom left, baseline BacSig/VirSig distribution in healthy males and females (GSE134080). (**D**) Bottom right, dynamic tuning of BacSig/VirSig during different stages of pregnancy (GSE108497). Pregnant women were divided into two groups, including the first 15 weeks of pregnancy (P01–15W) and the later phase of pregnancy (P16–40W).

In order to detect the abnormality of innate immunity in relevant diseases, it is crucial to know the features of VirSig and BacSig in a healthy population. A total of 209 healthy adults (21–82 years old) were included in the dataset GSE186507. It was clear that all of the subjects had a BacSig < 2, and only 6 subjects (2.9%) had a VirSig > 3 (Figure 2A). This indicated that an abnormal VirSig was rare and an abnormal BacSig was even rarer among healthy adults. In addition, the abnormal VirSig values in healthy adults could be due to asymptomatic respiratory viral infection, which is not uncommon. Nevertheless, it is recommended to follow up those subjects with abnormal VirSig or BacSig values. Another feature of this dataset is that no correlation was observed between age and either VirSig or BacSig (r = −0.08 for VirSig~Age and r = 0.12 for BacSig~Age). This indicated that the baseline VirSig and BacSig values were generally stable throughout healthy adulthood, and immunosenescence could not be reflected in these two parameters [32]. 

A related question is whether the maturation of the immune system during normal development can be reflected in the baseline VirSig or BacSig values [33]. A total of 77 healthy subjects with age spanning from 0.7 to 83.7 years old were included in the dataset GSE231409. For the convenience of analysis, these samples were divided into five age groups, including four development groups before 20 years old and one adult group after 20 years old. In this dataset, an upward trend was observed for BacSig during development, while no trend of change was observed for VirSig (Figure 2B). Due to the small sample size in each group, statistical significance was only reached for the BacSig comparison between the A15–20Y group and A00–05Y group or A10–15Y group (*p* < 0.05 for both). Nevertheless, the trend indicated increasing baseline BacSig during development and stabilization of the baseline VirSig at a very young age. Additionally, abnormal VirSig values (>3) were observed in four subjects across four age groups, indicating the non-correlation of an abnormal VirSig with age. Due to the shifting nature of the baseline BacSig values in each age group, an abnormal BacSig value should be defined based on the specific age group, which requires dedicated experiments with a much larger sample size for each age group. 

Apart from the age effect, the immune system is also different in males and females [34]. Dataset GSE134080 included 100 healthy adults (45 males and 55 females). In this dataset, all of the subjects had a BacSig < 2, while four subjects had a VirSig > 3 (all females, Figure 2C). The VirSig comparison between the two groups reached statistical significance (*p* = 0.036). This suggested that the BacSig values are generally stable among healthy adults, regardless of sex, while more outliers in the VirSig values may be expected in females. However, this was just an observation from one specific dataset. More independent experiments with larger sample sizes will be required for a better understanding of the sex effect on the baseline VirSig and BacSig values.

Another issue related to female adults is pregnancy, which also has a profound effect on the immune system [35]. A total of 187 healthy women at different stages of pregnancy were included the dataset GSE108497. In this dataset, all of the non-pregnant women had a BacSig < 2, while one of them had a VirSig > 3 (Figure 2D). Significant elevation of BacSig was evident during the first 15 weeks of pregnancy, and further elevation of BacSig was observed during the later phase of pregnancy. After the birth of the child (postpartum stage), the BacSig returned to the non-pregnant level. The *p*-values were <1.0 × 10^−7^ for all of the BacSig comparisons among the four groups, except for the comparison between the non-pregnant group and the postpartum group (*p* = 0.88). In comparison, the *p*-values were >0.05 for all of the VirSig comparisons among the four groups, indicating a non-significant adjustment of this immune component during pregnancy. Thus, BacSig/VirSig could reveal immune retuning during pregnancy.

### 3.3. Abnormality of VirSig and BacSig in Chronic Infection

**Figure 3:**
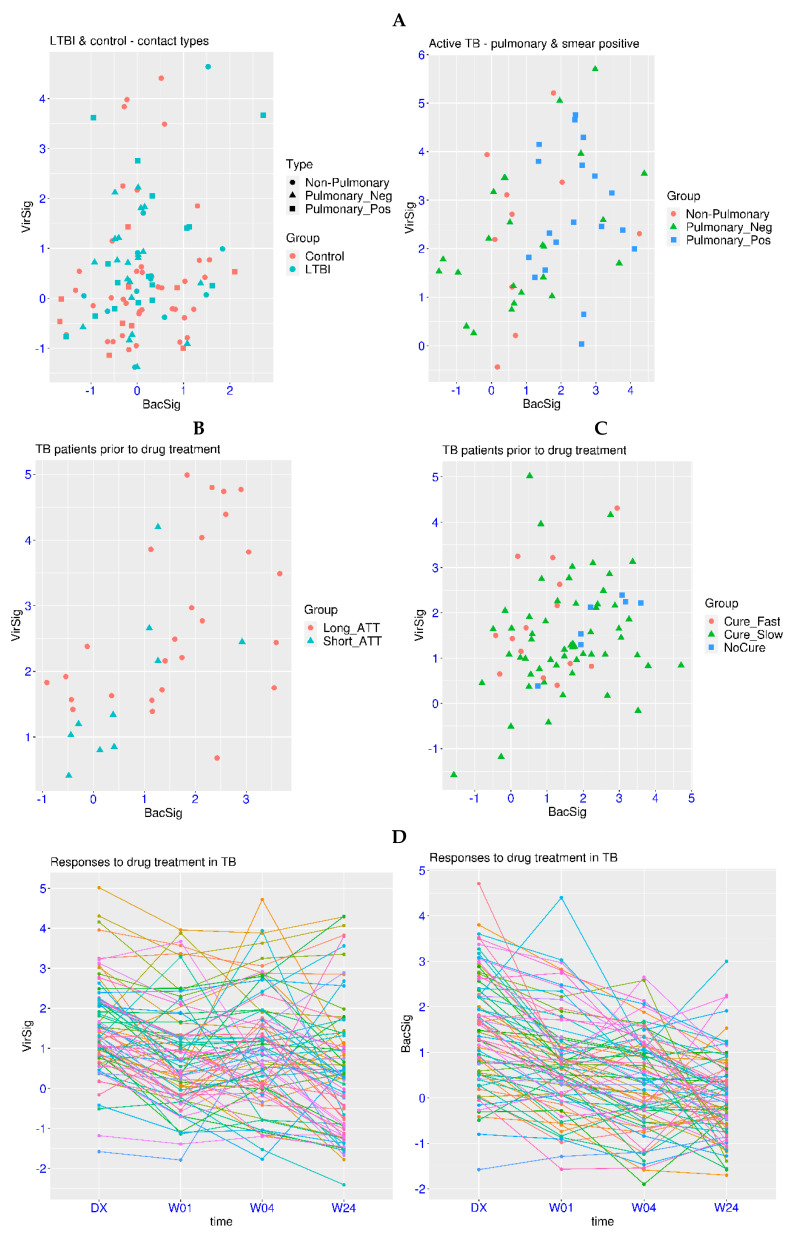
Serial monitoring of BacSig/VirSig in tuberculosis. (**A**) Top panel, features of BacSig/VirSig in negative controls, latent TB patients (LTBI) and three types of active TB patients at diagnosis (GSE107995). The LTBI and control subjects were indicated by the types of their contacts. (**B**) Middle left, features of BacSig/VirSig in two groups of TB patients prior to treatment (GSE157657). Short_ATT stands for standard anti-tuberculosis treatment regimen, while long_ATT stands for longer treatment regimen. (**C**) Middle right, features of BacSig/VirSig in three groups of TB patients prior to anti-tuberculosis treatment (GSE89403). Cure_Fast stands for cure within 4 weeks. Cure_Slow stands for cure between 8–24 weeks. NoCure stands for drug resistance. (**D**) Bottom panel, serial monitoring of BacSig/VirSig during the twenty-four weeks of medical intervention (GSE89403). The samples were taken at diagnosis (DX), week one (W01), week four (W04), and week twenty-four (W24) after the initiation of anti-tuberculosis treatment.

The above analyses have shown that abnormal VirSig and BacSig values are mainly observed in acute infection. Then, it is logical to ask whether the abnormality of VirSig and BacSig is involved in chronic infection. Tuberculosis is a chronic infection with the highest burden for the global medical system (https://www.who.int/, accessed on 20 March 2024). In the related dataset GSE107995, active TB patients were compared against negative controls and subjects with latent TB infection (LTBI). First, no significant differences were found for the comparison of the VirSig or BacSig between the latent TB group and the control group (*p* > 0.05 for both, Figure 3A). For the BacSig, one outlier (BacSig > 2) was observed in each group (50 subjects each). For the VirSig, four and three outliers (VirSig > 3) were observed in the control group and the latent TB group, respectively. In addition, one subject in the latent TB group had abnormality in both the BacSig and VirSig. It shall be noted that all of the subjects in these two groups were contacts of active TB patients, indicating possible hidden health problems, even in some of the control subjects. Interestingly, the two BacSig outliers in these two groups were both contacts of smear-positive pulmonary TB patients. Thus, continuous monitoring of those subjects with abnormal VirSig or BacSig values is warranted. 

For patients with active TB, abnormal VirSig (>3) or BacSig (>2) values were observed in 28 of the 53 patients (52.8%, Figure 3A). The *p*-values were <1.0 × 10^−7^ for the BacSig or VirSig comparison between the active TB group and the latent TB group, indicating the significant activation of VirSig and BacSig during the transition from latent TB to active TB. Additionally, compared to half of the controls having a VirSig < 0, only one patient with active TB had a VirSig < 0, indicating the significant upward shift of VirSig in the majority of the patients with active TB. 

Diverse features of VirSig and BacSig activation were also observed in different types of active TB patients. Among the 10 non-pulmonary TB patients, 2 BacSig outliers (20%) and 4 VirSig outliers (40%) were observed, including 1 patient with abnormality in both the BacSig and VirSig. Among the 24 smear-negative pulmonary TB patients, 5 BacSig outliers (20.8%) and 7 VirSig outliers (29.2%) were observed, including 3 patients with abnormality in both the BacSig and VirSig. In contrast, among the 19 smear-positive pulmonary TB patients (the most infectious type), 12 BacSig outliers (63.2%) and 8 VirSig outliers (42.1%) were observed, including 5 patients with abnormality in both the BacSig and VirSig. Overall, the proportion of BacSig outliers was much higher in the smear-positive pulmonary TB subgroup compared with the other two subgroups. Thus, distinctive immune profiles across different subgroups of active TB and patient heterogeneity within each subgroup could be reflected in the VirSig and BacSig values.

In the same study described above, longitudinal data on nine LTBI progressors was available. Among the nine patients, one had an abnormal VirSig value at baseline and was diagnosed with active TB in the second visit (VirSig further increased). Another patient had abnormal BacSig and VirSig values at baseline and was diagnosed in the second visit with normalized BacSig and VirSig values. The third patient had normal VirSig and BacSig values at baseline, followed by an abnormal BacSig value in the second visit, and a normalized BacSig value but an abnormal VirSig in the third visit (diagnosed). The fourth patient had simultaneous spikes of VirSig and BacSig values in the third visit and was diagnosed in the fourth visit with reduced VirSig and BacSig values. Thus, activation of VirSig or BacSig could proceed the diagnosis for some TB patients, and longitudinal monitoring of VirSig and BacSig could enhance our understanding of disease progression in TB. It could also facilitate the early detection of patients who may benefit from immediate intervention.

One of the major challenges for TB management is the understanding of the treatment response. In the related dataset GSE157657, patients with different treatment response were examined. First, one BacSig outlier (BacSig = 2.06) and two VirSig outliers (VirSig > 3) were observed within the control group (38 subjects). The *p*-values were 1.6 × 10^−5^ and 4.4 × 10^−11^ for the BacSig or VirSig comparison between the control group and the TB patient group, confirming the significant activation of VirSig and BacSig in TB. Among the 12 BacSig outliers in the patient group (36 TB patients, Figure 3B), only one patient was cured with a standard treatment regimen (short_ATT), and the other 11 patients all underwent longer treatment or even encountered drug resistance (long_ATT). A similar trend was observed for the VirSig. However, 7 of those TB patients has abnormality in both the BacSig and VirSig, making it difficult to evaluate the association of abnormal BacSig and VirSig with the treatment response. 

In another related dataset GSE89403, patients with different treatment response were also examined. First, no outliers were observed for either BacSig or VirSig within the control group (21 subjects). The *p*-values were 3.2 × 10^−8^ and 2.0 × 10^−5^ for the BacSig or VirSig comparison between the control group and the TB patient group. Among the 26 BacSig outliers in the patient group (78 TB patients, Figure 3C), only 2 patients were cured within 4 weeks (Cure_Fast), and the other 24 patients all underwent longer treatment (Cure_Slow) or even encountered drug resistance (NoCure). For the VirSig, there were only nine outliers in this cohort, including three in the Cure_Fast group and six in the Cure_Slow group. Overall, BacSig was more informative for the understanding of the treatment response in this cohort. The consistent feature in these two datasets was that an abnormal BacSig was associated with a longer treatment course or drug resistance. 

Serial monitoring of the treatment response is also important for complex diseases like TB. In the dataset GSE89403 described above, TB patients were sampled at diagnosis and three time points after the initiation of treatment. The decreasing trend was clear for the BacSig (Figure 3D). The number of BacSig outliers decreased from 26 at diagnosis to 11 at week 1, 5 at week 2 and 3 at week 24. The *p*-value was 1.9 × 10^−4^ for the BacSig comparison between diagnosis and week one, and the *p*-value was also 1.9 × 10^−4^ for the BacSig comparison between week one and week twenty-four. From diagnosis to week twenty-four, there was a decrease of 1.47 for the BacSig. On the other hand, the trend of the change was not as clear for the VirSig. The number of VirSig outliers were 9 at diagnosis, 7 at week 1, 6 at week 4 and 7 at week 24. A more subtle change was revealed by statistical analysis. The *p*-value was 0.01 for the VirSig comparison between diagnosis and week one, and the *p*-value was 0.026 for the VirSig comparison between week four and week twenty-four. From diagnosis to week twenty-four, there was a decrease of 1.11 for the VirSig. Overall, normalization of both the VirSig and BacSig was observed during the twenty-four weeks of the medical intervention. However, persistent abnormality in the VirSig was observed in at least four of the cured patients, which might explain the unresolved hyper-inflammation in some of the cured patients. In addition, persistent abnormality in the BacSig was observed in one of the patients who encountered drug resistance. Thus, serial monitoring of VirSig and BacSig could enhance our understanding of full recovery and drug resistance in TB patients. 

**Figure 4:**
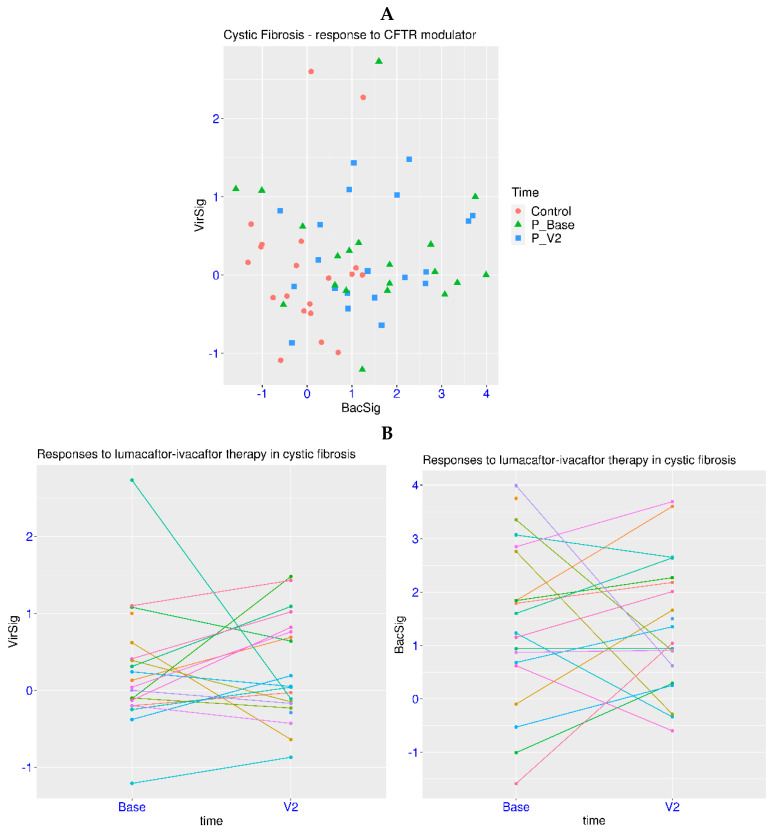
Serial monitoring of BacSig/VirSig in cystic fibrosis. (**A**) Upper panel, features of BacSig/VirSig in healthy controls, and CF patients before and after CFTR treatment (GSE124548). (**B**) Lower panel, changes in BacSig and VirSig in CF patients at the two time points (GSE124548). Base stands for baseline (prior to treatment). V2 stands for the second visit (six months later).

Cystic fibrosis is a genetic disease accompanied by chronic infection, which is different from TB, with an infectious pathogen as the causal factor. Modern treatments for CF mainly target the cystic fibrosis transmembrane conductance regulator (CFTR) [36]. In a related dataset GSE124548, CF patients undergoing treatment with a CFTR modulator were monitored for six months. First, there were no outliers for either the BacSig or VirSig in the control group (20 subjects, Figure 4A). There were also no VirSig outliers in the patient groups. On the other hand, there were 6 BacSig outliers among the 20 patients prior to treatment, and there were 7 BacSig outliers 6 months after the initiation of treatment. The *p*-values were >0.05 for the VirSig comparisons among the three groups. For the BacSig comparison, the *p*-value was >0.05 between the two patient groups, but it was <0.0025 for the comparison of the control group with either patient group. This indicated that a significant portion of CF patients had an abnormality in the BacSig, regardless of the treatment.

More details could be revealed when the patients were tracked for the changes in the VirSig and BacSig after the treatment (Figure 4B). First, the changes in the VirSig were less relevant because all of the samples had a VirSig < 3. On the other hand, a shifting of the BacSig status was observed in several patients. Three patients had decreased BacSig (from > 2 to < 2) after the treatment, while five patients had increased BacSig (from <2 to >2). In addition, two patients retained outlier status after the treatment. Overall, the treatment did not show a significant effect on reducing the BacSig abnormality in the CF patients as a group. Due to the long time span between the two sampling points, the exact dynamic change in the BacSig was unclear. Further details may be revealed with an improved sampling strategy.

### 3.4. Abnormality of VirSig and BacSig in Autoimmune Diseases

**Figure 5:**
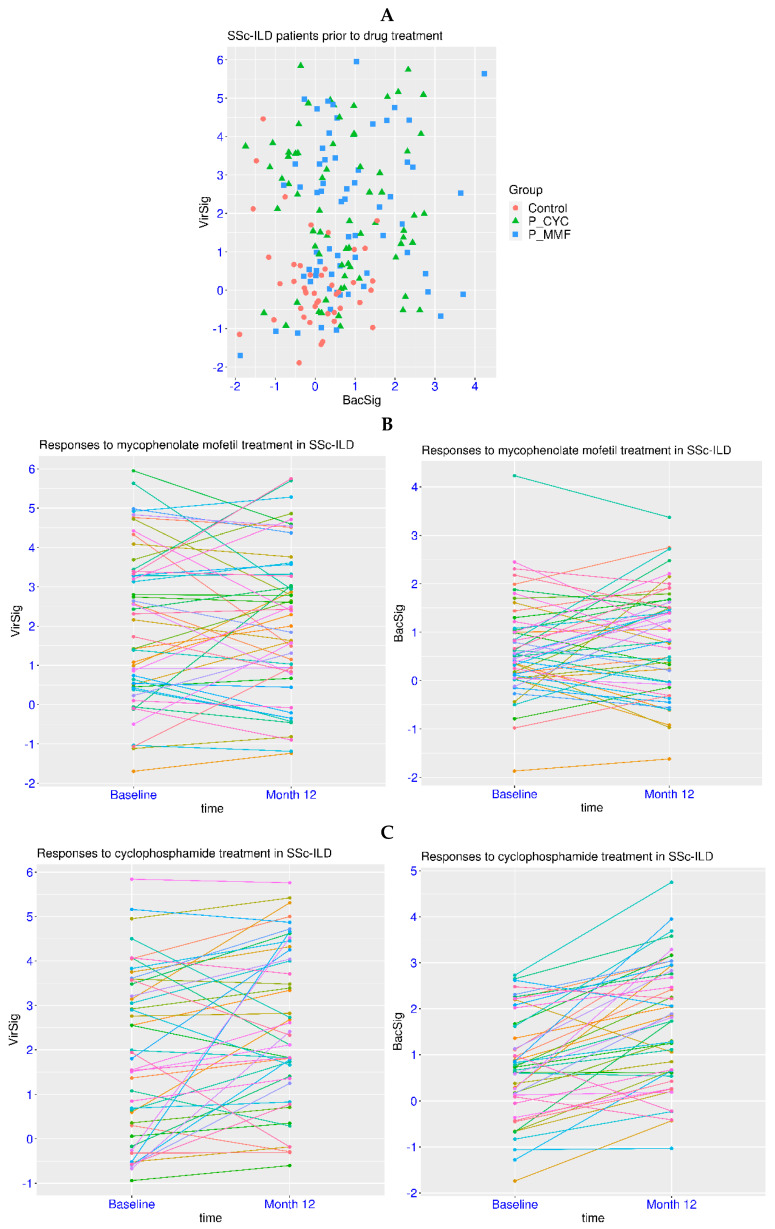
Serial monitoring of BacSig/VirSig in SSc-ILD. (**A**) Top panel, features of BacSig/VirSig in healthy controls and SSc-ILD patients prior to CYC or MMF treatment (GSE181228). P_CYC and P_MMF indicated the two patient groups before treatment. (**B**) Middle panel, changes in BacSig and VirSig in SSc-ILD patients before and after the MMF treatment (GSE181228). (**C**) Bottom panel, changes in BacSig and VirSig in SSc-ILD patients before and after the CYC treatment (GSE181228). The two sampling points were baseline (before treatment) and month twelve (after the initiation of treatment).

Dysregulation of innate immunity has been reported for autoimmune diseases such as SLE and systemic sclerosis. Systemic sclerosis is frequently manifested as interstitial lung disease (SSc-ILD). In a related dataset GSE181228, patients with SSc-ILD were examined for the treatment effect of two immunosuppressants, cyclophosphamide (CYC) and mycophenolate mofetil (MMF). First, there were no subjects with a BacSig > 2 and only two subjects with a VirSig > 3 within the group of 45 healthy controls (Figure 5A). In contrast, 26 of the 69 patients in the CYC treatment group had a VirSig > 3, and 20 of the 65 patients in the MMF treatment group had a VirSig > 3 (prior to treatment for both). The *p*-value was <1.0 × 10^−5^ for the comparison of the VirSig between the control group and either of the two patient groups, and it was >0.05 for the VirSig comparison between the two patient groups. In addition, there were 15 and 11 patients with a BacSig > 2 in the CYC and MMF treatment groups, respectively. The *p*-values were <0.0035 for the comparison of the BacSig between the control group and either of the two patient groups, and it was >0.05 for the BacSig comparison between the two patient groups. Overall, this indicated the abnormality of the VirSig and BacSig in a significant portion of patients with SSc-ILD.

Next, the effect of the drugs on the immune profile of the patients was evaluated in the paired samples. For the MMF treatment, the *p*-values were >0.05 for the comparison of either the VirSig or BacSig before and after the treatment (Figure 5B). Both an increase and a decrease in the VirSig and BacSig were observed, indicating no significant change in the VirSig and BacSig abnormalities due to the MMF treatment. For the CYC treatment, the *p*-value was 0.0056 for the comparison of the VirSig before and after the treatment, and it was 6.1 × 10^−8^ for the comparison of the BacSig at the two time points (Figure 5C). The mean values of the VirSig and BacSig were shifted upwards after the treatment (+0.63 for VirSig and +0.92 for BacSig), indicating increased VirSig and BacSig abnormalities due to the CYC treatment. This was consistent with the worse tolerability and toxicity profile of CYC observed in clinical trials [37]. Thus, the assessment of VirSig and BacSig could assist in the evaluation of the treatment effect on the immune profile in relevant diseases.

**Figure 6:**
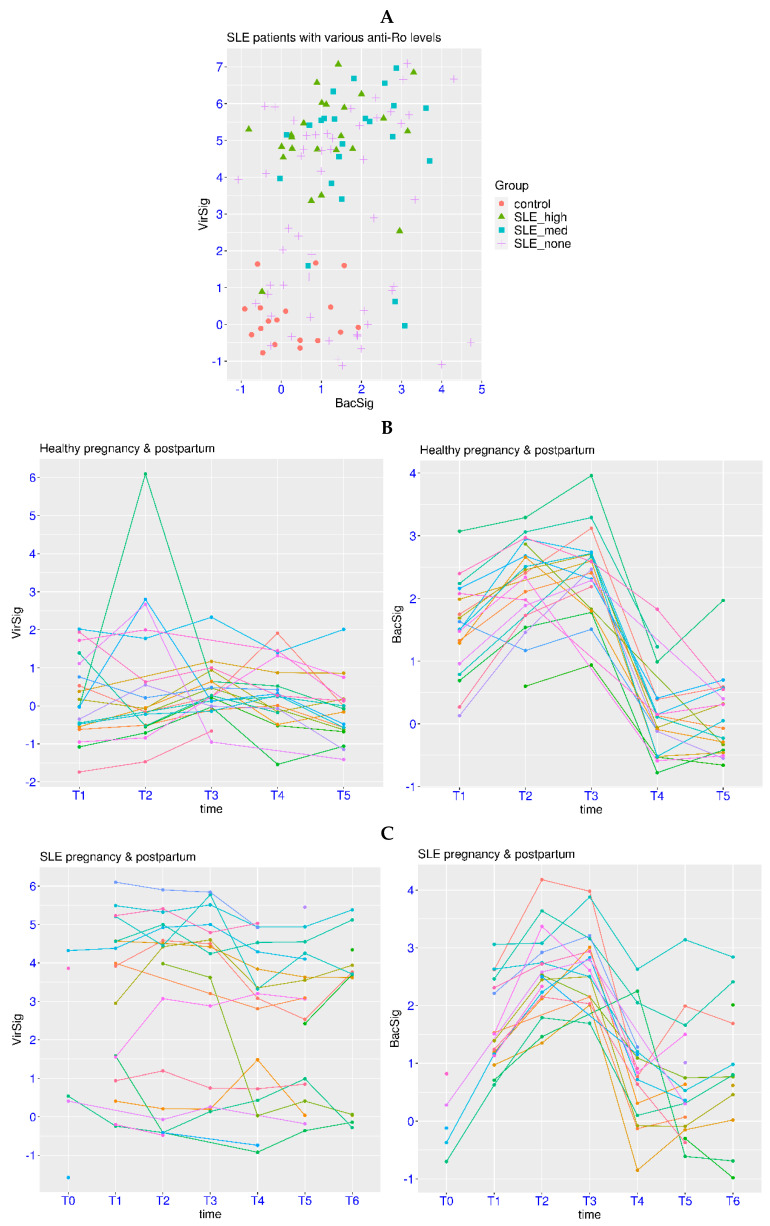
Serial monitoring of BacSig/VirSig in SLE. (**A**) Top panel, features of BacSig/VirSig in healthy controls and SLE patients with various anti-Ro levels (GSE72509). SLE_high stands for patients with high anti-Ro level. SLE_med stands for patients with medium anti-Ro level. SLE_none stands for patients with non-detectable anti-Ro. (**B**) Middle panel, dynamic changes in BacSig and VirSig in healthy women at different stages of pregnancy (GSE235508). (**C**) Bottom panel, dynamic changes in BacSig and VirSig in SLE patients at different stages of pregnancy (GSE235508). T1 through T3 stand for the three trimesters of pregnancy. T0 stand for non-pregnant stage. T4 through T6 stand for the postpartum time points (6 weeks, 6 months and 12 months).

The mechanism of persistent immune dysregulation is also of vital importance. In a related dataset GSE72509, SLE patients with various anti-Ro levels were examined. In this dataset, all of the 18 control subjects had a BacSig < 2 and a VirSig < 2 (Figure 6A). In contrast, most of the SLE patients with detectable anti-Ro autoantibody had a VirSig > 3. More specifically, 22 of the 24 patients (91.7%) with a high anti-Ro level (SLE_high) had a VirSig > 3, and 20 of the 23 patients (87.0%) with a medium anti-Ro level (SLE_med) had VirSig > 3. The *p*-value was <1.0 × 10^−7^ for the comparison of the VirSig in the control group against the VirSig in either the SLE_high group or SLE_med group, and it was >0.05 for the comparison of the VirSig between the SLE_high and SLE_med groups. In addition, 26 of the 52 patients (50.0%) with no detectable anti-Ro (SLE_none) also had a VirSig > 3. The *p*-values were <0.003 for the comparison of the VirSig in the SLE_none group against VirSig in any of the other three groups. This indicated that anti-Ro might make a direct contribution to the abnormal VirSig in some SLE patients, while factors other than anti-Ro could also contribute to the abnormal VirSig in SLE patients. 

Abnormality in the BacSig was also observed in a significant portion of SLE patients in this cohort. The BacSig was >2 in 5 patients (20.8%) in the SLE_high group, 10 patients (43.5%) in the SLE_med group, and 17 patients (32.7%) in the SLE_none group. The *p*-value was 3.1 × 10^−4^ for the BacSig comparison between the control group and SLE_med group, and it was 0.0045 for the BacSig comparison between the control group and SLE_none group. Overall, the prevalence of abnormality in VirSig and BacSig was high in this cohort of SLE patients. In conjunction with SLE-specific tests such as autoantibody tests, the assessment of VirSig and BacSig could enhance the understanding of pathogenesis and facilitate patient stratification for SLE. 

Since the prevalence of SLE is highly biased toward females, pregnancy is a common concern among SLE patients [38]. In the related dataset GSE235508, pregnant women with or without SLE were examined throughout different stages of pregnancy. Consistent with the dataset GSE108497 in Figure 2D, significant elevation of BacSig was observed in all three trimesters of healthy pregnancy (more so for T2 and T3, Figure 6B). Eight, twelve and fourteen healthy subjects had a BacSig > 2 at T1, T2 and T3, respectively, while no healthy subjects had a BacSig > 2 at T4 and T5 (postpartum time points). In contrast, only one healthy subject had a VirSig > 3 at T2 throughout all five stages. This confirmed that the BacSig was retuned during healthy pregnancy. For SLE patients, significantly more abnormality was found in the BacSig and VirSig throughout different stages of pregnancy (Figure 6C). Six, fifteen and fifteen SLE patients had a BacSig > 2 at T1, T2 and T3, respectively. In addition, three, one and three SLE patients had a BacSig > 2 at T4, T5 and T6, respectively. On the other hand, the majority of SLE patients had a VirSig > 3 at every stage, and the VirSig was relatively stable in most of the SLE patients from T1 to T6. Overall, the additive effect of immune dysregulation was observed in SLE patients during pregnancy. Serial monitoring of this immune profile is recommended to understand its association with an adverse pregnancy outcome (APO). 

## 4. Discussion

One of the major challenges of infectious diseases is antibiotic resistance. To alleviate this problem, antibiotics should not be prescribed to those who do not need them. A common clinical scenario is the judgement of viral or bacterial infection in case of fever and other symptoms [39]. This work demonstrated that patients with acute bacterial infection generally had a BacSig > 2, while healthy people and those with inactive colonization generally had a BacSig < 2. Therefore, it is not suggested to prescribe antibiotics to those patients with a BacSig < 2, because the symptoms are unlikely to originate from bacterial infection. On the other hand, certain patients with viral infection might also have a BacSig > 2, and certain patients with bacterial infection might also have a VirSig > 3. In addition, factors other than infection may also lead to the activation of the BacSig or VirSig [40]. Thus, a simple judgment of infection types based on a BacSig > 2 (for bacterial infection) or a VirSig > 3 (for viral infection) may result in a significant portion of misclassification. This kind of approach has been attempted in previous works and should be cautioned against [41]. 

Acute infection can leave a mark on the innate immune system for some patients. Longitudinal monitoring of the VirSig and BacSig post acute infection can help to understand the source of the problems in the PAS. Generally, the VirSig and BacSig shall return to the normal level at the convalescence stage and thereafter. Sustained activation of the VirSig or BacSig could be an indication of unresolved inflammation and should be taken care of. This was demonstrated in Ebola survivors and CFS. It might also be relevant to long-COVID [42]. For more precise and personalized health monitoring, it is suggested to have multiple measurements of the VirSig and BacSig at the healthy stage as a personalized baseline, which may facilitate the detection of moderate abnormality deviated from individual baseline post acute infection. 

Although the VirSig and BacSig were derived from acute infection, abnormality of the VirSig and BacSig has been demonstrated in chronic infection and autoimmune diseases. A significant portion of active TB patients had an abnormality in the VirSig or BacSig, while latent TB patients were indistinguishable from negative controls. In addition, some patients had transient activation of the BacSig or VirSig prior to the diagnosis of active TB. This indicated that the VirSig and BacSig played important role in the pathogenesis of active TB. Furthermore, an abnormal BacSig was associated with longer treatment courses in two independent cohorts. In addition, persistent activation of the VirSig was observed in some cured patients, which might be associated with unresolved hyper-inflammation in a portion of cured patients. Thus, the exact roles that the BacSig and VirSig play in the pathogenesis and treatment response of active TB warrants further investigation.

The evaluation of the treatment effect was also demonstrated for CF and SSc-ILD. In CF, it was shown that an abnormal BacSig was observed in some patients and the CFTR modulator did not change the overall BacSig abnormality in the patient cohort. In SSc-ILD, abnormality of the VirSig and BacSig was observed in some patients and the MMF treatment did not change the overall BacSig abnormality in the cohort. More importantly, the CYC treatment led to an increase in the overall BacSig and VirSig abnormalities. This again highlighted the value of longitudinal monitoring of the VirSig and BacSig in relevant diseases. Moreover, sampling at multiple time points may further improve our understanding of the dynamics of innate immunity post drug treatment.

As for SLE, detectable anti-Ro autoantibody was associated with an abnormal VirSig in SLE patients. This might explain the mechanism of persistent VirSig activation in SLE and other related diseases. In addition, an abnormal VirSig was also observed in some patients negative for anti-Ro, suggesting that other factors, including autoantibodies, may be the source of VirSig activation in those patients. Tracing back the source of the VirSig and BacSig abnormality may lead to a targeted strategy for the treatment of SLE and related diseases. Another issue for SLE is the complication during pregnancy. The additive effect of VirSig and BacSig activation was observed during the pregnancy for SLE patients. The relevance of this feature to the APO deserves further investigation. 

Although this work was mainly focused on RNA-Seq data, the findings are expected to be applicable to other gene expression platforms. For examples, activation of the BacSig was evident in bacterial meningitis (an acute bacterial infection, GSE40586, microarray, Supplementary Figure S1) [43], and activation of the VirSig was evident in HIV-positive subjects (a chronic viral infection, GSE28686, microarray, Supplementary Figure S2) [44]. Another relevant issue is whether abnormal VirSig and BacSig can be observed in tissues other than whole blood. Surprisingly, activations of both the VirSig and BacSig were found in psoriasis skin (an autoimmune disease, GSE66511, RNA-Seq, Supplementary Figure S3) [45]. 

This work is mainly limited by the availability of high-quality data, especially studies with a longitudinal experimental design. The high-throughput nature of transcriptome datasets also makes it challenging to derive quantitative models of a few pre-selected genes. On another note, this work is also limited to the assessment of two well-known pathways of innate immunity. The immune system of humans is highly complex. This work is not intended to cover all the aspects of innate immunity, not to mention adaptive immunity. The purpose of this work is to help dissect the heterogeneity in infectious and autoimmune diseases. This work is only a proof of concept. Dedicated experimental works with low-throughput platforms available in clinical settings will be required in the future. Nevertheless, the low cost of the assay will make it possible to conduct sampling at much shorter intervals and reveal finer details of the dynamic change in innate immunity.

## 5. Conclusions

In this work, a 2D gene model for the quantitative assessment of systemic innate immunity was proposed. Through the analysis of 2168 samples in 17 independent transcriptome datasets, it was repeatedly demonstrated that healthy subjects generally had a BacSig < 2 and a VirSig < 3. Simultaneous assessment of the VirSig and BacSig can assist decision making on antibiotic prescription as well as patient stratification and prognosis in relevant diseases. Longitudinal assessment of the VirSig and BacSig can assist the monitoring of recovery from acute infection as well as normal development and pregnancy. Longitudinal assessment of the VirSig and BacSig can also facilitate the understanding of the pathogenesis and evaluation of treatment response in relevant diseases. 

## Figures and Tables

**Table 1:** Transcriptome datasets for the examination of the abnormality of VirSig and BacSig in health and disease.

Accession ID	Condition	Sample Numbers	Abnormality	Ref
GSE161731	Acute infection Adults	19 controls, 17 viral infection, 23 bacterial infection	VirSig and BacSig	[12]
GSE176262	Acute infection Adults	30 controls, 48 viral infection, 35 bacterial infection	VirSig and BacSig	[13]
GSE158163	GAS infection Children	16 controls, 10 GAS infection, 5 GAS colonization	BacSig	[14]
GSE72946	Leptospirosis Adults	4 controls, 16 acute phase, 13 convalescence phase	BacSig	[15]
GSE143549	Ebola survivors Adults	33 controls, 26 Ebola survivors	VirSig and BacSig	[16]
GSE98139	CFS Adolescence	19 controls, 28 CFS patients	BacSig	[17]
GSE186507	Ageing	209 healthy adults	VirSig (rare)	[18]
GSE231409	Development	77 healthy subjects	Increasing BacSig	NA
GSE134080	Gender Adults	55 females, 45 males	VirSig (rare)	[19]
GSE108497	Pregnancy Female adults	23 controls, 147 pregnant women 17 postpartum women	Mainly BacSig + VirSig (rare)	[20]
GSE107995	Tuberculosis Adults Subtypes	50 negative controls, 50 latent TB patients, 53 active TB patients	BacSig and VirSig	[21]
GSE157657	Tuberculosis Adults Treatment effect	38 negative controls, 36 active TB patients Before treatment	BacSig and VirSig	[22]
GSE89403	Tuberculosis Adults Treatment effect	21 negative controls, 78 active TB patients 4 time points	BacSig and VirSig	[23]
GSE124548	Cystic fibrosis Adults Treatment effect	20 negative controls, 20 CF patients 2 time points	BacSig	[24]
GSE181228	SSc-ILD Adults Treatment effect	45 controls, 134 patients (×2)	VirSig and BacSig	[25]
GSE72509	SLE Adults Subtypes	18 controls, 99 SLE patients	VirSig and BacSig	[26]
GSE235508	SLE Adults Pregnancy	19 controls (×5) 18 SLE patients (×6)	VirSig and BacSig	[27]

## Data Availability

All of the datasets analyzed in the current work are publicly available. The transcriptome datasets are available in the GEO (gene expression omnibus, https://www.ncbi.nlm.nih.gov/geo/, accessed on 20 March 2024). For more details, please refer to the Section 2 and Table 1.

## Supplementary Materials

The following supporting information can be downloaded at https://www.mdpi.com/article/10.3390/biomedicines12050969/s1, Figure S1: Patterns of VirSig and BacSig activation in bacterial meningitis (BM); Figure S2: Patterns of VirSig and BacSig activation in methcathinone users; Figure S3: Patterns of VirSig and BacSig activation in psoriasis skin; Table S1: P values for the group comparisons described in the main text.
