# Supplementary material for: Quantitative and Longitudinal Assessment of Systemic Innate Immunity in Health and Disease Using a 2D Gene Model
Source: Biomedicines. 2024 Apr 27;12(5):969. doi: 10.3390/biomedicines12050969 (PMC11117654; doi:10.3390/biomedicines12050969)
Supplement: Supplementary file 1 [file biomedicines-12-00969-s001.zip › Supplementary figures.pdf]

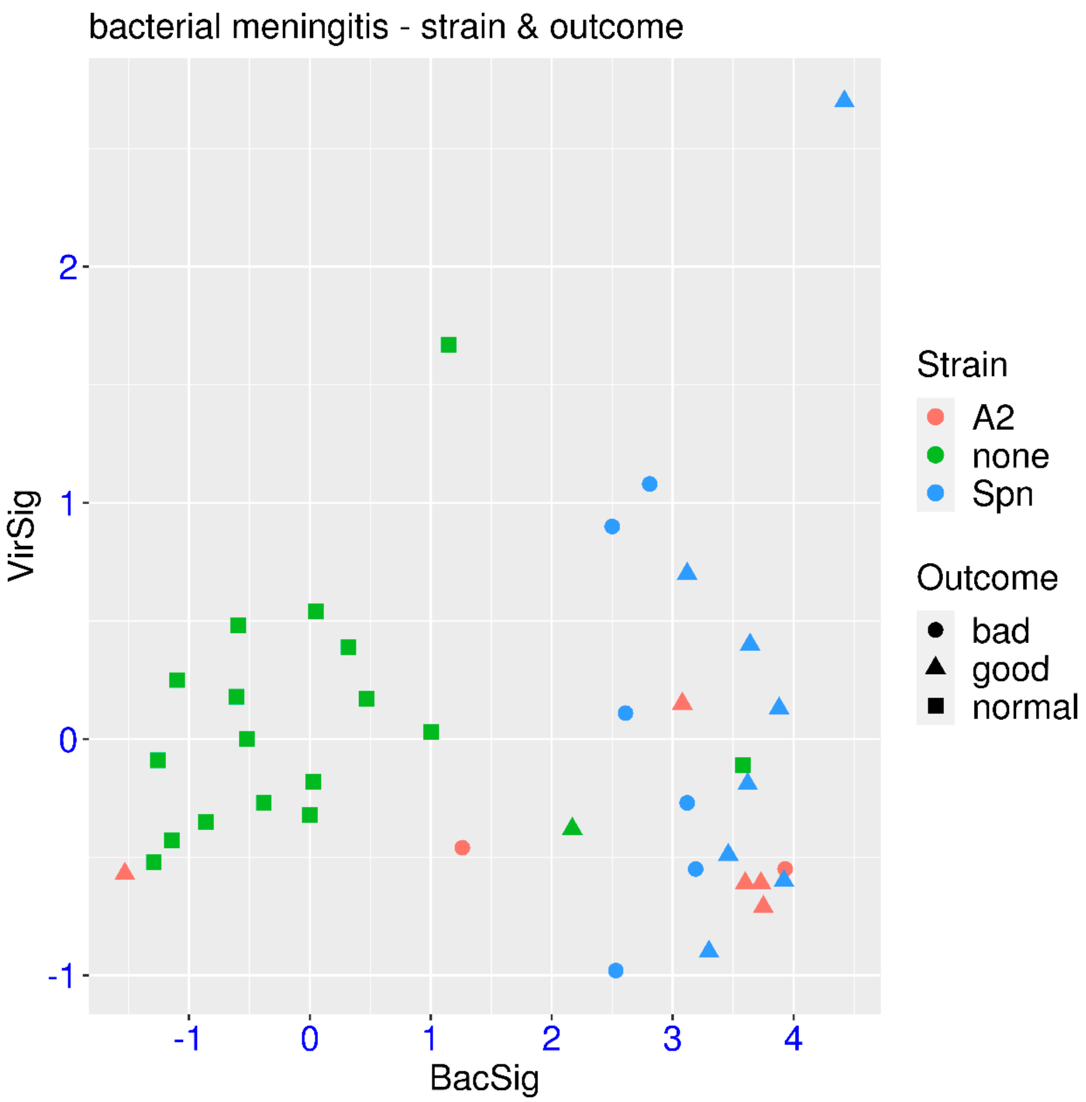

Supplementary Figure S1 Patterns of VirSig and BacSig activation in bacterial meningitis (BM). The patients with BM were compared with healthy controls (GSE40586, whole blood). The bacterial strains included Streptococcus pneumoniae (Spn) and A2. The outcome of the patients was also indicated in the figure.

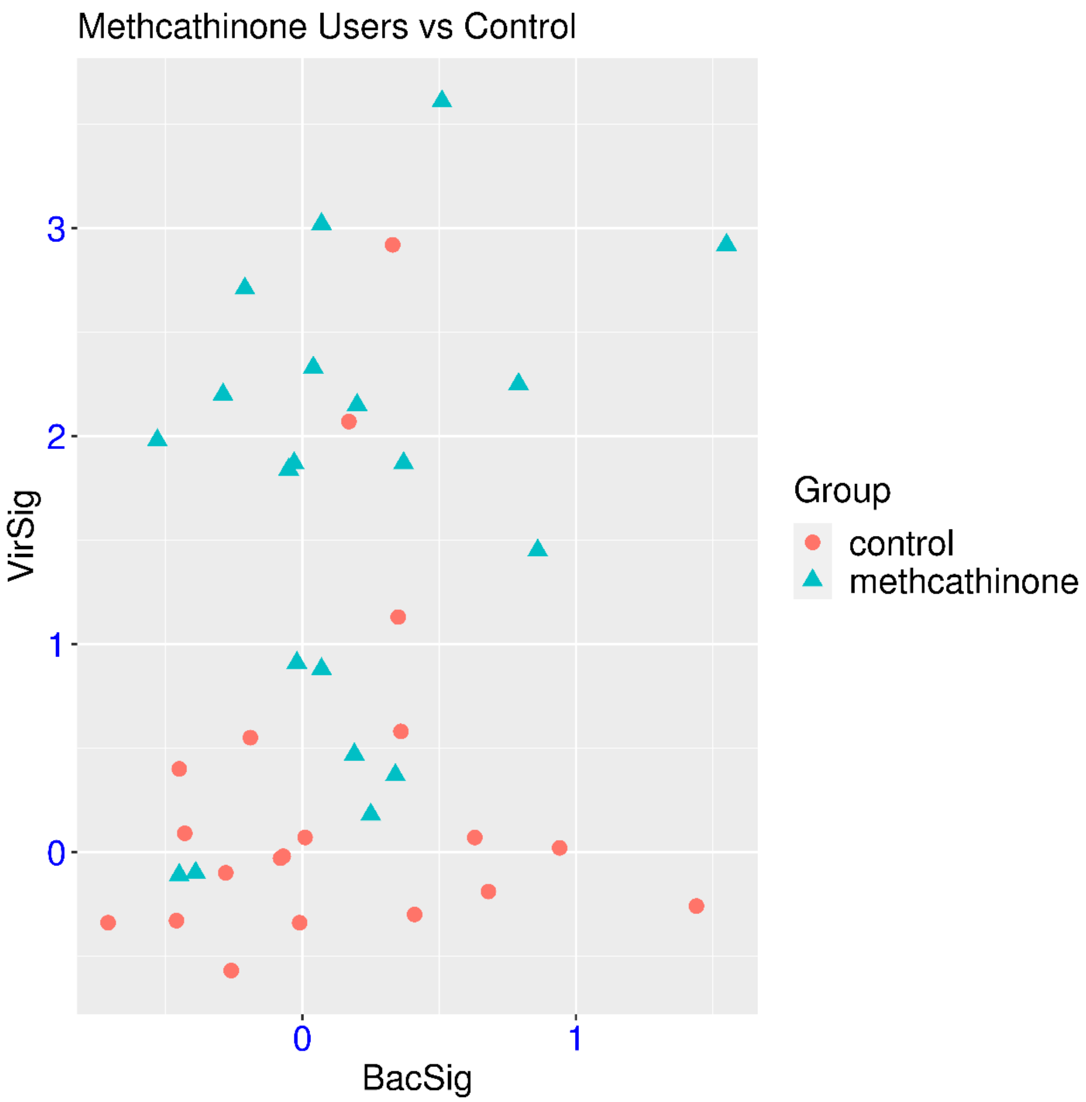

Supplementary Figure S2 Patterns of VirSig and BacSig activation in methcathinone users. The drug users compared with healthy controls (GSE28686, whole blood). It shall be noted that 15 of the drug users were positive for HIV.

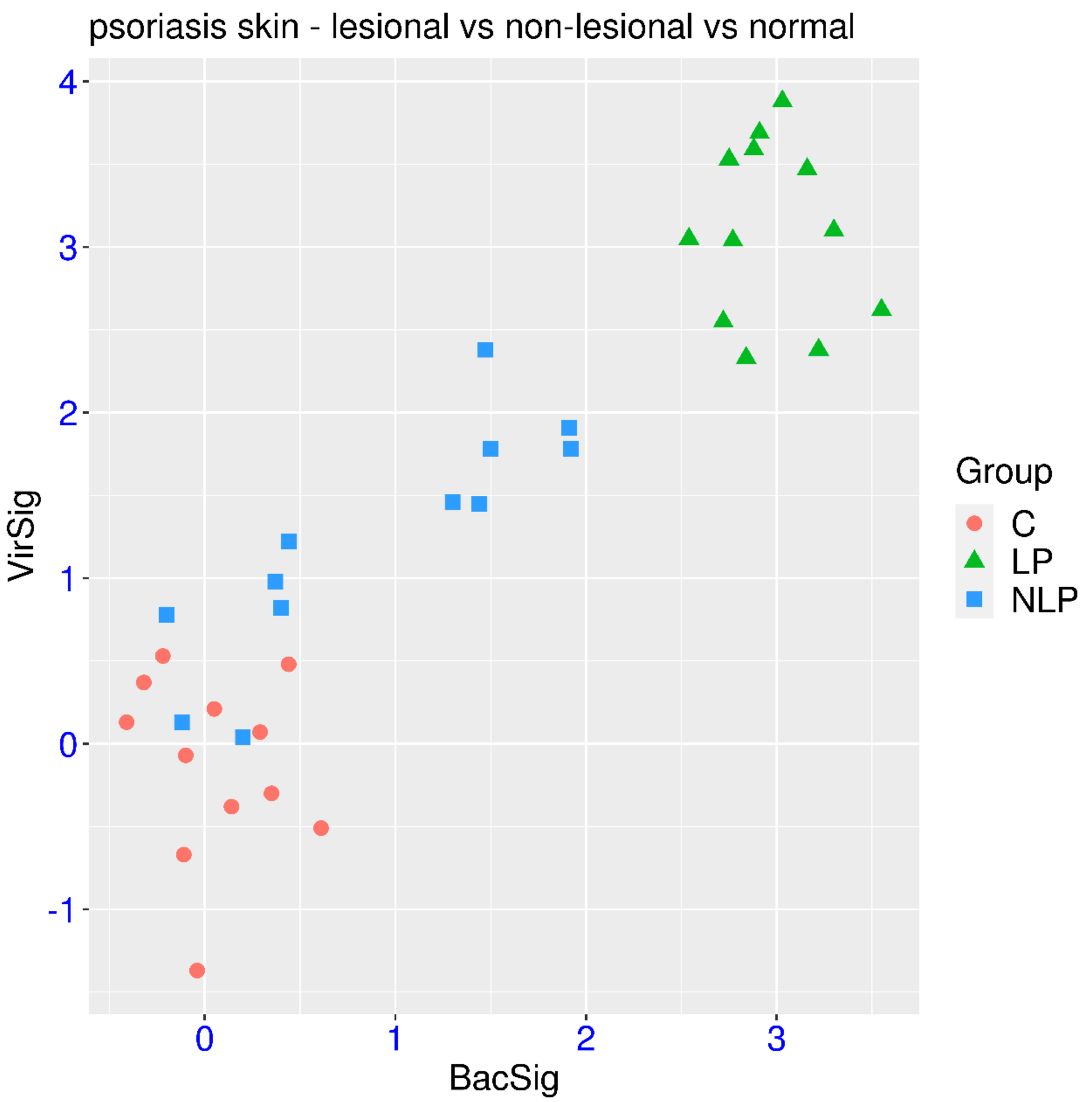

Supplementary Figure S3 Patterns of VirSig and BacSig activation in psoriasis skin. Patients with psoriasis were compared with healthy controls (GSE66511, skin). Both lesional and non-lesional skins were included in the study. .
